# Morphological Comparison of the Maxillary Arch in Buccal and Palatal Canine Impaction among Asian Population of Gujarati Origin: A Hospital-Based Study

**DOI:** 10.3390/healthcare10050939

**Published:** 2022-05-19

**Authors:** Falguni Mehta, Mayank Jain, Swati Verma, Sakeenabi Basha, Renuka A. Patel, Rahul Trivedi, Harshik A. Parekh, Valai Kasim Shakeel Ahmed, Mohammad Khursheed Alam, Anil Kumar Nagarajappa, Pratibha Taneja

**Affiliations:** 1 Department of Orthodontics and Dentofacial Orthopaedics, Government Dental College and Hospital, Ahmedabad 380016, India; drfalgunimehta1@gmail.com (F.M.); mkjain164@gmail.com (M.J.); renu.patel216.rp@gmail.com (R.A.P.); dr.rahultrivedi@gmail.com (R.T.); harshikp@yahoo.com (H.A.P.); 2 Division of Orthodontics and Dentofacial Deformities, Centre for Dental Education and Research, All India Institute of Medical Sciences, New Delhi 110029, India; 3 Department of Community Dentistry, Faculty of Dentistry, Taif University, Taif 21944, Saudi Arabia; sakeena@tudent.edu.sa; 4 Department of Orthodontics, Ragas Dental College and Hospital, Chennai 600119, India; drshakeelahmed_vk@yahoo.com; 5 Orthodontics, Preventive Dentistry Department, College of Dentistry, Jouf University, Sakaka 72345, Saudi Arabia; 6 Center for Transdisciplinary Research (CFTR), Saveetha Institute of Medical and Technical Sciences, Saveetha Dental College, Saveetha University, Chennai 602117, India; 7 Department of Public Health, Faculty of Allied Health Sciences, Daffodil lnternational University, Dhaka 1230, Bangladesh; 8 Oral Medicine & Radiology, Department of Oral & Maxillofacial Surgery & Diagnostic Sciences, College of Dentistry, Jouf University, Sakaka 72345, Saudi Arabia; dr.anil.kumar@jodent.org; 9 Department of Public Health Dentistry, Sudha Rusatgi College of Dental Science and Research, Faridabad 121001, India; pratibhataneja3@gmail.com

**Keywords:** maxillary impacted canine, MIC, buccal impaction, palatal impaction, palatal depth, morphology, inter-molar width

## Abstract

Aim: To estimate the differences in the maxillary arch morphology in buccal and palatal canine impaction in an Asian population of Gujarati origin. Methodology: An institutional ethics committee’s approval was acquired before the commencement of this study. Sixty subjects were enrolled in the study. Thirty subjects (20 females and 10 males) had a maxillary impacted canine either buccal or palatal and thirty control group participants were selected aged 13 to 18 years who sought orthodontic treatment at the tertiary health care center in Ahmedabad, Gujarat, in western India. Routine pre-treatment radiographs and dental plaster models with good anatomic details were recorded. Measurements of the inter-molar width, palatal depth, arch length, sum of the mesio-distal width of the upper incisors, and available arch space were recorded from prepared orthodontic study models using digital vernier calipers with an accuracy of 0.01 mm and brass wire. The ratio of palatal depth to inter-molar width (Ratio 1), arch length to inter-molar width (Ratio 2), and width of the maxillary incisors to available arch space (Ratio 3) were also secondarily calculated. Data were analyzed using Statistical Package for Social Sciences (SPSS) version 21, IBM Inc. The normality of the data was assessed by the Shapiro–Wilk test. As the data was found to be normally distributed, bivariate analyses were also performed (one-way ANOVA test, Bonferroni post hoc correction). The level of statistical significance was set at a *p*-value less than 0.05. Results: The comparison of the inter-molar width, palatal depth, arch length, sum of the mesio-distal width of the upper incisors, available arch space, Ratio 1, Ratio 2, and Ratio 3 among controls and subjects with buccal and palatal canine impaction showed overall significant differences in the inter-molar width, palatal depth, arch length, sum of the mesio-distal width of the upper incisors, and available arch space when compared using one-way ANOVA as *p* < 0.05. Ratios 1, 2, and 3 also showed significant differences between the buccal and palatal canine impaction. Conclusion: An inadequate arch length (*p* < 0.0001) and a higher degree of crowding with reduced available arch space (*p* < 0.0001) may be considered as early risk factors for buccal maxillary canine impaction. An inadequate inter-molar width (*p* < 0.0001), and an increased palatal depth (*p* < 0.0001) with a clinically reduced mesiodistal width of the sum of maxillary incisors may be considered as risk factors for palatal maxillary canine impaction in an Asian population of Gujarati origin.

## 1. Introduction

The eruption of teeth is a sequential, systematic, and physiological procedure taking place in the human body. Pathologically, tooth impaction can be described as a situation where a fully developed tooth stays lodged within either soft or hard tissue past its natural eruption phase [1]. Impaction-related eruption disorder is a common occurrence in the events leading up to the transition from primary to permanent dentition. [2,3]. Patil S et al. reported a prevalence of tooth impaction of 16% in Asians of Indian origin [4]. Apart from the third molars, the maxillary canines are the most susceptible, with a prevalence of 9.7% [4], far greater than the 0.2 to 9.0% documented in other ethnic groups [5,6,7]. After third molars, maxillary canines are the most commonly impacted teeth, with 1–2 percent of the population being affected [4,8,9,10]. As the maxillary canine tooth germ has a greater vertical distance to travel before reaching the final occlusal table, this adds time to the whole eruption process. Because of this, it’s more vulnerable to impaction [11,12]. Around the age of three years, the canine is located high in the maxilla, with its crown directed in a mesio-lingual direction [12]. It descends gradually, becoming erect as it moves until it reaches the level of the incisor root, where it assumes an almost vertical position. A preponderance of canine impactions occurs on or near the palatal side (70–85%) of the dental arch, while 15–30% occur on or near the buccal side of the dental arch [12]. Quite frequently, however, it erupts with considerable mesial inclination. During such a developmental course, various factors may lead to its impaction. Canine impaction is most frequently caused by the insufficient resorption of the predecessor tooth, a traumatised tooth bud, a disruption in the order of tooth eruption, an insufficient amount of accessible space within the maxillary arch, tooth germ deflection from its original position, and premature closure of the apical foramen, as well as in association with any craniofacial anomalies. Buccal or palatal divergence of maxillary impacted canines from a regular eruptive pathway is commonly reported. According to the research so far, the etiological hypothesis for these two possible divergences differs significantly [13]. Becker et al. posited the guidance theory for palatal MIC as a result of local predisposing conditions in their research, such as congenitally missing teeth, supernumerary teeth, or odontomes [14]. Peck & Peck emphasised that a palatally displaced canine, PDC, was genetically determined [15]. The presence of anomalous tooth structure correlates with genetic predilection. He stated that overall, 33% of individuals with PDC are born with congenitally missing teeth. Brin et al. [16] observed that 65 percent of PDC demonstrated anomalous structural variation in relation to the lateral incisor, whereas Bishara et al. [17] revealed that congenitally missing maxillary lateral incisor occurred and was accompanied by palatal maxillary impacted canines (Palatal MIC) 2.5 times more than in the general population. When it pertains to buccal maxillary impacted canines (Buccal MIC), inadequate arch length and crowding are more typically implicated [9]. In addition, McConnell et al. demonstrated that the transverse maxillary arch width is altered in canine impaction, especially in the premaxillary region of a patient [18]. They also determined a distinct etiology for palatal MIC. They found a reduced maxillary width as a local mechanical factor and suggested that patients with palatal canine impaction would be advantageous to treat with maxillary expansion as the main interventional treatment option [18]. Schindel R et al. [19] observed increased maxillary arch width, whereas Langberg B found no correlation between canine impaction and maxillary transverse width [20]. The disparity in maxillary transverse measurements, according to Schindel and Duffy [19], enhances the risk of canine impaction. In contrast, Al-Nimri et al. [21] discovered that participants with palatal MIC have larger maxillary transverse arch dimensions. Studies were conducted in the past to demonstrate a link between the location of the maxillary impacted canine and the morphology of the maxillary arch. However, most of them were performed among Caucasians and other ethnic groups. Jain et al. also confirmed that there was a need to perform more research on maxillary canine impaction prevalence as per ethnicity, race, and origin of the population groups [22]. There was a dichotomy in the available literature. As a result, more investigation was required to determine whether the location of canine impaction correlates with the morphology of the maxillary arch. To the best of our knowledge, no previous study has examined a link between the location of the maxillary impacted canine and the morphology of the maxillary arch in the Asian population of Gujarati origin. Thus, the present hospital-based study was designed as per STROBE guidelines [23] to determine the differences in the maxillary arch morphology in buccal and palatal maxillary impacted canines among an Asian population of Gujarati origin aged between 13 and 18 years.

## 2. Materials and Methods

### 2.1. Settings

An institutional ethics committee’s approval was acquired before the commencement of this study. (IEC Number: April 2014/32). This was a cross-sectional, hospital-based study conducted at a tertiary health care center. Between April 2014 and April 2015, this study was conducted using subjects aged 13 to 18 years who sought orthodontic treatment at the tertiary health care centre in Ahmedabad, Gujarat, in western India. Power calculation and sample size estimation were performed using nMaster’s software (version 2, Christian Medical College, Vellore, India). Considering the clinically meaningful mean difference in AL/IMW × 100 of palatally and bucally erupted canines with an effect size of 1.11, a minimum sample size of 58 was found to be sufficient for α of 5% and Power of 95%. Hence, a total of 60 samples were selected in order to anticipate the possible dropouts.

### 2.2. Participants

**Figure 1:**
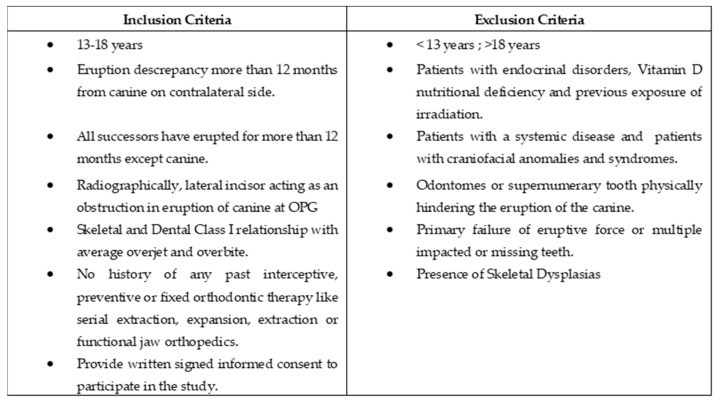
Inclusion and Exclusion criteria.

Sixty subjects were enrolled in the study, thirty of whom had a maxillary impacted canine, either buccal or palatal (20 females and 10 males), and thirty of whom served as age-matched controls for the study. Clinical photographs, lateral cephalograms, panoramic X-rays, and Clark’s method intraoral periapical radiographs were used to determine the exact diagnosis [3] for each patient. The confirmatory diagnosis was made by looking for an eruption discrepancy exceeding 12 months from the canine on the contralateral side [24] and if the canine remained unerupted after all successors had erupted for more than 12 months [10]. The encroaching proximity of the lateral incisor with an unerupted canine was a positive finding in OPG [10,11], confirmed by the IOPA with Clark’s technique [3]. The additional criteria for inclusion were a Class I skeletal and molar relationship and a lack of prior exposure to orthodontic expansion, extraction, or fixed orthodontic treatment with an average overjet, overbite, and absence of crossbite. The exclusion criteria included patients with any endocrinal disorders like thyroid or pituitary deficiency, nutritional deficiency of Vitamin D, patients with previous exposure to irradiation, patients with a systemic disease, patients with craniofacial anomalies and syndromes, absence of advanced caries, odontomes or supernumerary teeth physically hindering the eruption of the canine or primary failure of eruptive force, or multiple impacted or missing teeth were also excluded. Written consent was sought from all the participants and patients’ parents. An information sheet was also provided to them for their volunteer participation. The inclusion and exclusion criteria are summarized in Figure 1. The Maxillary impacted canine (MIC) group (*n* = 30) was divided into two subgroups: the Buccal Maxillary Impacted Canine (Buccal MIC) (*n* = 12), and the Palatal Maxillary Impacted Canine (Palatal MIC) (*n* = 18). A control group of 30 patients with a similar age range and inclusion and exclusion criteria (*n* = 30) was employed to compare with the MIC group.

### 2.3. Landmarks and Measurements

**Figure 2:**
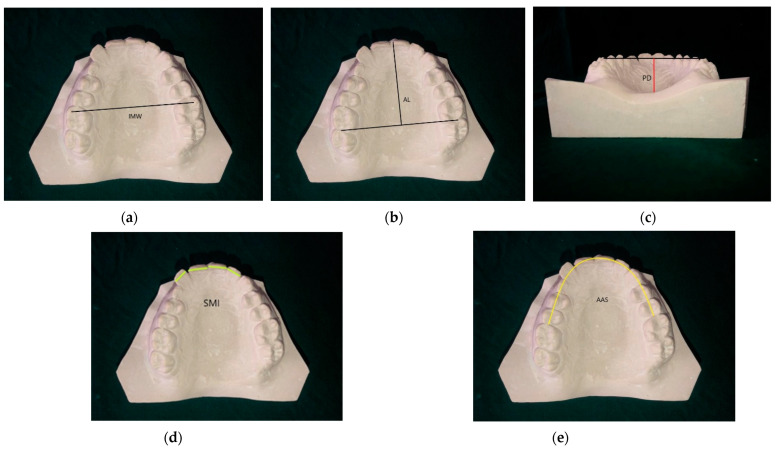
Maxillary Arch Measurements: (**a**) inter-molar width; (**b**) arch length; (**c**) palatal depth (**d**) sum of maxillary incisors; (**e**) available arch space.

**Figure 3:**
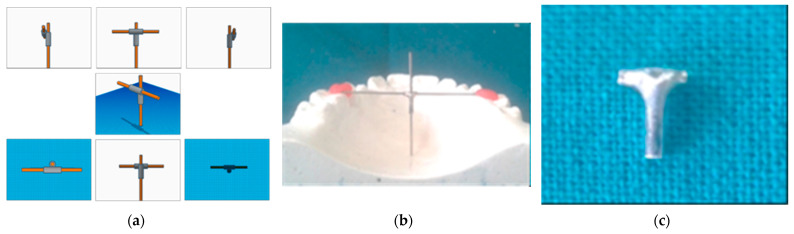
Palatal Depth Measurement Device: (**a**) 3D simulation views- right, front, left, oblique, top, rear, bottom; (**b**) Palatal Depth Measurement Device while taking measurement on study model; (**c**) Closer view of T- shaped assembly alone.

For each patient, pre-treatment dental plaster models with good anatomic details were obtained and preserved as a part of the routine procedure at our center. The maxillary arch measurements recorded in this study were: inter-molar width (IMW), arch length (AL), palatal depth (PD), the sum of the mesio distal width of upper incisors (SMI), and the available arch space (AAS). These measurements were made directly on the diagnostic model, considering landmarks as specified in Table 1 and Figure 2 [24]. The linear measurements were recorded with Digital Sliding Calipers (400-044 Eclipse Tools Electronic Digital Caliper, Alexandria, VA, USA) measuring to the nearest 0.01 mm and brass wire. The inter-molar width was measured between the mesiobuccal cusp tips of both first molars. The arch length was measured from the line joining the distal surfaces of both first molars to the mesial surface incisal edge of either central incisor in a sagittal direction. The available arch space was measured from the mesial surfaces of the right to the left first molars with the help of a brass wire. Palatal depth was measured by the Palatal Depth Measuring Device. This device was indigenous and fabricated with readily available materials in the medical setup. The schematic three-dimensional design is represented in Figure 3. It consisted of a T-shaped configuration assembly custom made using two small pieces of hollow 18-gauge surgical needle of predetermined length and an internal lumen size of 0.036′′. They were oriented perpendicular to each other. Both tubes were spot-welded, followed by soldering. Then, the T-shaped assembly was polished and finished. This assembly was used in conjunction with two removable stainless steel straight wires of 0.036′′ dimension. The ends of the horizontal wire were stabilised at the mesiopalatal cusps of the study model in a manner so that the T-shaped assembly lies in line with the mid-palatine suture, then a second SS wire was dropped through the vertical lumen till the base of the palate. The upper terminal opening was marked with a marker. The vertical distance between the palatal end of the wire and the marked terminal point was recorded as the palatal depth.

Kim et al. [24] demonstrated that the ratio of linear variables plays a significant role in statistically calculating and comparing the relative geometry mean. According to his observations, calculating ratios would be an effective way to evaluate the morphology of the maxillary arch between the study cohorts. For instance, the ratio of arch length to inter-molar width (Ratio 1) may be used to compare the maxillary arch morphological features between the experimental groups. In the same way, the ratio of palatal depth to inter-molar width (Ratio 2) may assess the relative form of the palate, and the ratio of the sum of the four maxillary incisor widths and available arch space (Ratio 3) can estimate whether the maxillary arch had adequate eruption space for the eruption of the canine or not. As a result, we preferred to calculate ratios 1, 2, and 3 among the selected subjects of an Asian population of Gujarati origin in this study. Firstly, one Orthodontist, well-trained and calibrated under an experienced Orthodontist, identified the landmarks, recorded the linear measurements and calculated the Maxillary Arch Ratios manually over the study models, followed by the repetition of 6 randomly selected study models by the same Orthodontist and second Orthodontist after 4 weeks.

### 2.4. Data Analysis

Statistical analysis was performed using Statistical Package for Social Sciences (SPSS) (version 21 IBM Corp., 2015, Virginia, USA). Descriptive data has been presented for each variable. Descriptive statistics such as frequencies, the mean and standard deviation for continuous variables were reported. To assess the normality of the data, the Shapiro–Wilk test was used. Based on the normal distribution, bivariate analyses were performed using one-way ANOVA test followed by Bonferroni post hoc correction for pairwise comparison. Statistical significance was set at *p*-value < 0.05.

## 3. Results

### 3.1. Sample Demographics of Buccal, Palatal MIC and Controls

There were a total of 60 study subjects, out of which 30 subjects were recruited in control groups and the other 30 subjects were considered as cases having buccal MIC (*n* = 12) or palatal MIC (*n* = 18). As evident in Table 2, the mean age among subjects with buccal MIC was found to be 15.6 ± 1.8 years and among subjects with palatal MIC it was found to be 15.8 ± 1.6 years. Among controls, the mean age was calculated to be 15.7 ± 2.2 years. Out of the 12 subjects with buccal MIC, 8 were females and 4 were male subjects, and among the 18 subjects with palatal MIC, 12 were females and 6 were males. Among controls, 16 were females and 14 were males. Bilateral buccal and palatal MIC was seen in 4 and 5 subjects, respectively, whereas unilateral buccal and palatal MIC was seen in 8 and 13 subjects, respectively. On the left side, buccal and palatal MIC was appreciated among 7 and 11 subjects, respectively, whereas on the right side it was appreciated in 5 and 7 subjects, respectively.

### 3.2. Comparison among Buccal, Palatal MIC and Control Group

Table 3 depicted the intergroup comparison as follows. The comparison of IMV, PD, AL, SMI, AAS and Ratio 1, Ratio 2 and Ratio 3 among controls and subjects with buccal and palatal MIC showed overall significant differences in the IMV, PD, AL, SMI, AAS, Ratio 1, Ratio 2, and Ratio 3 when compared using one-way ANOVA as *p* < 0.05. Post hoc comparison showed significantly more IMW, PD, and SMI in buccal MIC or controls as compared to palatal MIC or controls. AAS was found to be significantly more in controls followed by buccal and palatal MIC, whereas AL was found to be significantly more in palatal MIC and controls as compared to buccal MIC. Ratio 1 was found to be significantly more in palatal MIC followed by buccal MIC and controls.

To evaluate intra-examiner and inter-examiner reliability, the initial Orthodontist and another trained, calibrated Orthodontist randomly selected six study models (10% of the total sample size) and recorded all measurements after 4 weeks interval. The Cronbach’s Alpha coefficient of pooled measurements was calculated and summarized as 0.89 exhibiting good intra-examiner and inter-examiner agreement.

## 4. Discussion

Inter-molar width was observed to be significantly higher in the Buccal MIC group than in the Palatal MIC group in the present study. This is in concurrence with the study by Kim et al. [24] who found a mean inter-molar width of 54.06 ± 5.87 mm in a Buccal MIC group and 52.16 ± 5.89 mm in a palatal MIC group with *p* = 0.003, whereas Yan et al. [25] found no difference in the inter-molar widths between patients with Buccal MIC and palatal MIC. Contrary to this study, Hong et al. [26], Langberg et al. [20], Al-Nimri et al. [21], Anic-Milosevic et al. [27], and Fattahi H et al. [28] found no difference in the inter-molar widths between patients with palatal MIC and controls. The palatal vault is steeper in the palatal MIC group as compared to the Buccal MIC group. Contrary to this study, Fattahi H et al. [28] and Anic-Milosevic et al. [27] found no differences between the palatal vault depth of patients with palatal MIC and controls. This implies that the palatal shape is narrower and deeper in the palatal MIC group than in the Buccal MIC group and the control group. The steeper palatal vault suggests the presence of bilaterally more vertically inclined palatal shelves. Due to the increased vertical orientation of palatal shelves, it is envisaged that the tooth germ will traverse a greater distance through the maxillary bone until complete occlusion. As a result, canine impaction occurs. A late developing dentition and hypo-developed lateral incisors are also contributing factors to palatal MIC. It is evident that a lateral incisor that is underdeveloped will not be able to adequately perform the function of guidance in this situation [14]. In the palatal MIC group, the lateral incisor is also expected to be displaced. As a corollary, the distance between the radicular tip of the lateral incisor and the canine root tip may be larger than that of the buccal MIC group. The potential of palatal impaction with a deep palatal vault can be explained by the same assumption as early emergence of the lateral incisor or deferred canine migration [24]. Leonardi et al. and Tepedino et al. established a correlation between the sella turcica and palatally displaced canines [29,30]. Tepedino et al. observed that there was a significant association between the reduced length of sella bridging and palatally displaced canines [30]. The rationale for this logical association was the sharing of the anterior wall of the sella with the dental lamina, a common genetic embryological origin from the neural crest cells, and the same stood for maxillary arch too [31,32].

The arch length for the Buccal MIC group is shorter than both the controls and the palatal MIC group and this difference among the three groups is highly significant statistically. In concordance with this study, Kim et al. [24] observed a statistically significant difference in the mean arch length between the buccal MIC group and the palatal MIC group of 39.76 ± 2.65 mm and 40.80 ± 2.17 mm (*p* = 0.042). Stellzig et al. [33] conducted a study on 63 patients with 83 impacted canines which concluded that an arch deficiency was found only in 18% of patients with palatal MIC, whereas there was an arch-length deficiency in 46% of the patients with buccal MIC.

Ratio 1, that is, arch length to inter-molar width for the Buccal MIC group was found to be 70.85 ± 4.41 in the present study. Several authors found the same findings as the present study in their respective studies. Kim et al. [24] who observed that the ratio of arch length ×100/ inter-molar width to be 73.39 ± 4.47 in the Buccal MIC group and 78.46 ± 4.60 in the palatal MIC group. The difference in the sum of the incisors between the palatal MIC group and controls was found to be statistically significant (Table 3). This observation may be linked to a peg shaped or missing lateral incisor, as has been suggested by Becker et al. [13] and Brin et al. [16] in their research studies. Mercuri et al. [34] further confirmed that a relatively higher percentage of shape and size differences of the maxillary lateral incisors were likely to show its association with palatal canine impaction than buccal canine impaction. However, no statistically significant difference was found between all the three groups. Similar to present study, Langberg et al. [20] found statistically significant tooth size reductions associated with palatal MIC as compared to controls, whereas Al–Nimri et al. [21] found no difference in the mesio-distal width of the maxillary teeth of patients with palatally impacted teeth and controls. Available arch space in the Buccal MIC group was found to be 71.73 ± 4.11 mm, in the palatal MIC group it was 75.73 ± 4.63 mm, and in the control group it was found to be 78.36 ± 4.15 mm. Contrary to this, Mercuri et al. [34] reported that patients with palatal MIC had a lower degree of dental arch crowding in comparison to the controls. The ratios 1, 2, and 3 demonstrated a lengthier, constricted maxillary arch with steeper palatal vault depth and adequate available arch space for the eruption of permanent dentition as compared to the buccal MIC group in an Asian population with Gujarati origin.

### 4.1. Clinical Implication

Our results reflected that the morphological traits of the maxillary arch may be utilized as a risk predictor for the diagnosis of maxillary impacted canine. The localization of the canine was performed with a clinical examination and conventional radiography like OPG, Occlusal radiograph, right-angle technique, and SLOB’s technique [35]. The results of this study pertaining to maxillary arch dimensions would act as adjunctive to confirm and predict buccal or palatal maxillary canine impaction in the early diagnosis phase. With an insight into how these characteristics deviate from normal morphology and the early diagnosis of canine eruption disturbances, the clinician can better explain the circumstances to the patients’ parents and can investigate and plan the most appropriate interceptive or ortho-surgical intervention [36]. Moreover, as a diagnostic predictor these measurements can also prevent the unnecessary CBCT exposure to the patients. According to guidelines of the American Dental Association Council on Scientific Affairs [37], the clinicians are encouraged to schedule and perform CBCT exams based on their professional judgement, with published CBCT guidelines confirmed as well as specific patient clinical conditions and requirements. By utilising CBCT only when it is absolutely essential, it is possible to limit orthodontic patients’ unnecessary exposure to ionising radiation. While CBCT can be an extremely useful diagnostic tool for orthodontists, it’s use should be case-specific, with the practitioner able to justify the need of CBCT [37]. As this demonstrates, when it comes to orthodontic CBCT, proceed with caution and clinical reasoning judgement. Cases of maxillary impacted canine that are simple, mild to moderately complicated, can be easily diagnosed and treated using standard routine diagnostic and treatment procedures such as conventional radiographs and maxillary arch measurements [37,38].

### 4.2. Limitation

The authors advocate and advise that the current study be expanded into a prospective translational study with a larger sample size to establish norms for maxillary arch dimensions in individuals with buccal and palatal MIC that will represent the demographic variables and traits of this population group as well as be compared to other ethnic groups. Digital models may also be considered in accordance with the cost-benefit ratio.

## 5. Conclusions

The evidence from this study concluded that there is a link between the site of maxillary canine impaction and the morphology of the maxillary arch in an Asian population of Gujarati origin as follows:

- Inadequate arch length (*p* < 0.0001) and a higher degree of crowding with reduced available arch space (*p* < 0.0001) may be considered as early risk factors for buccal maxillary canine impaction.
- An inadequate inter-molar width (*p* < 0.0001), and increased palatal depth (*p* < 0.0001) with a clinically reduced mesiodistal width of the sum of maxillary incisors, may be considered as a risk factor for palatal maxillary canine impaction in an Asian population of Gujarati origin.

## Figures and Tables

**Table 1:** Maxillary impacted canine characteristics, landmarks and measurements employed in this study as described by Kim et al. [24].

**Details of Maxillary Impacted Canine Characteristics, Landmarks and Measurements Employed in This Study**	
Type	Unilateral or Bilateral
Side	Right or left
Location	Buccal or Palatal
**Landmarks and Measurements Employed**	
Inter-molar width; IMW (mm)	The transverse distance between the mesiobuccal cusp tips of both first molars.
Palatal depth; PD (mm)	The perpendicular height measured from the line joining the mesiopalatal cusp tips of both first molars to the palatal surface in vertical direction along the midline.
Arch Length; AL (mm)	The perpendicular distance, measured from the line joining the distal surface of both first molars to mesial surface incisal edge of either central incisor in saggital direction.
Sum of Maxillary incisors; (mm)	The sum of the mesiodistal widths of all four maxillary incisors.
Available arch space; AAS (mm)	The space measured through adjoining teeth from the mesial surfaces of the right to the left first molars with the help of brass wire.
Ratio 1 AL × 100/IMW	This value signifies Maxillary arch shape.
Ratio 2 PD × 100/IMW	This value signifies palatal vault shape.
Ratio 3 SMI × 100/AAS	This value indicates whether or not the maxillary permanent dentition had adequate space to erupt.

**Table 2:** Descriptive Statistics of Study groups.

Variables		Maxillary Impacted Canine (MIC) *			Controls (30)
		Buccal (12)	Palatal (18)	Total (30)	
Age (Mean ± SD) (Years)		15.6 ± 1.8	15.8 ± 1.6	15.7 ± 1.9	15.7 ± 2.2
Sex *n* (%)	Females Male	8 (26.66%) 4 (13.33%)	12 (40%) 6 (20%)	20 10	16 (53.33%) 14 (46.60%)
Origin	Gujarati	12	18	30	30
Bilateral: Unilateral		4:8 (33.3%:66.6%)	5:13 (27.7%:72.2%)	9:21	
Left: Right		7:5 (50.8%:41.6%)	11:7 (61.1%:38.8%)	18:12	

* MIC, Maxillary Impacted Canine.

**Table 3:** Statistical comparison of maxillary arch measurements between the groups. The measurements recorded were IMW, Inter-molar width; PD, Palatal depth; AL, Arch Length; SMI, Sum of Maxillary Incisors; AAS, Available Arch Space and the Ratio 1,2, and 3.

**Parameters**	**Group**	**Mean**	**SD**	**Std. Error**	**Minimum**	**Maximum**	**Anova** ***p*-Value**	**Post Hoc**
IMW	Buccal side	51.04	2.93	0.535	45.52	57.08	<0.0001 *	1.3 > 2
	Palatal side	48.22	2.65	0.484	43.75	59.07		
	Controls	50.42	2.61	0.477	46.25	57.47		
PD	Buccal side	19.32	1.86	0.340	16.12	25.03	<0.0001 *	1.3 > 2
	Palatal side	21.33	2.08	0.380	15.62	25.93		
	Controls	19.28	1.96	0.358	15.01	24.02		
AL	Buccal side	35.93	1.71	0.313	30.76	39.73	<0.0001 *	2.3 > 1
	Palatal side	39.70	2.18	0.398	35.08	43.82		
	Controls	39.62	1.98	0.361	34.53	43.37		
SMI	Buccal side	30.94	2.47	0.452	26.01	36.77	0.014	1.3 > 2
	Palatal side	29.62	2.14	0.391	26.10	33.57		
	Controls	31.26	2.13	0.389	26.93	36.73		
AAS	Buccal side	71.73	4.11	0.750	63.94	79.55	<0.0001 *	3 > 2 > 1
	Palatal side	75.57	4.63	0.845	67.67	86.06		
	Controls	78.36	4.15	0.758	71.67	89.69		
RATIO 1 AL × 100/IMW	Buccal side	70.85	4.41	0.804	60.98	80.92	<0.0001 *	2 > 3 > 1
	Palatal side	82.47	5.42	0.989	69.54	91.24		
	Controls	78.71	4.88	0.892	68.71	91.84		
RATIO 2 PD × 100/IMW	Buccal side	37.90	3.11	0.568	33.34	47.25	<0.0001 *	2 > 1.3
	Palatal side	44.28	4.18	0.763	33.90	53.89		
	Controls	38.35	4.46	0.813	28.05	49.43		
RATIO 3 SMI ×100/AAS	Buccal side	43.86	3.74	0.682	38.11	51.68	<0.0001 *	1 > 2.3
	Palatal side	39.33	3.73	0.681	32.70	47.92		
	Controls	39.89	1.87	0.341	36.01	43.45		

* Statistical Significance, *p* < 0.05.

## Data Availability

Anonymized data are available on request from the corresponding author and with permission of the participants in the study. The data presented in this study are not publicly available due to ethical reasons and to protect the privacy of participants.

## Abbreviations

MIC, Maxillary Impacted Canine; IMW, Inter-molar width; PD, Palatal depth; AL, Arch Length; SMI, Sum of Maxillary Incisors; AAS; Avaliable Arch Space.
